# Are the Adaptogenic Effects of Omega 3 Fatty Acids Mediated via Inhibition of Proinflammatory Cytokines?

**DOI:** 10.1155/2012/209197

**Published:** 2011-10-10

**Authors:** Joanne Bradbury, Lyndon Brooks, Stephen P. Myers

**Affiliations:** ^1^NatMed-Research Clinical Trials Unit, Southern Cross Plant Science, Southern Cross University, P.O. Box 157, Lismore, NSW 2480, Australia; ^2^Division of Research, Southern Cross University, P.O. Box 157, Lismore, NSW 2482, Australia

## Abstract

The study was undertaken to estimate the size of the impact of n-3 fatty acids in psychological stress and the extent to which it is mediated via proinflammatory cytokines. Structural equation modeling (SEM) was used to analyze data from 194 healthy Australians. Biomarkers used were erythrocyte polyunsaturated fatty acids (docosahexaenoic acid (DHA) and arachidonic acid (AA)), *ex-vivo* stimulated secretion of proinflammatory cytokines (interleukins (IL-1 and IL-6), and tumor necrosis factor (TNF)). Stress was measured with the perceived stress scale (PSS-10), found to comprise three factors: Coping (items 4, 7, 5), Overwhelm (2, 10, 6 and 8), and Emotional (1, 9 and 3). This modeling demonstrated that the effects of DHA on coping are largely direct effects (0.26, *t* = 2.05) and were not significantly mediated via the suppression of proinflammatory cytokines. Future modeling should explore whether adding EPA to the model would increase the significance of the mediation pathways.

## 1. Introduction

The prevalence of chronic stress is widespread in all Western countries. Long-term exposure to stress increases the risk of cardiovascular disease [1–3] and is a predictor of future psychiatric disorders [4–6]. Stress is a multidimensional physiological and psychological phenomenon. Metabolic, haemostatic, and immune functions are altered to optimize the chances of surviving a threat to homeostasis. The well-known “fight or flight” response immediately diverts all resources required for survival. Blood pressure and blood glucose are raised to provide for enormous immediate energy expenditure; clotting elements, and cell-mediated immunity are activated to preserve blood loss and protect against microbial invasion. These transient changes are adaptive, such that a tear in the skin has the resources for quick repair [7]. Stress induces a vigilant, proinflammatory systemic, or metabolic state that is adaptive as long as it is acute. 

Stress response is quickly extinguished if the stress is cognitively appraised as nonthreatening. Stress appraisal is multifaceted, taking into account the threat of the stressor and the capacity to cope with it [8]. Based on the appraisal model, the individual responses to stress will be a function of the primary cognitive appraisal (of the immediate environmental stressor) offset by the secondary appraisal (of available resources for coping). A net imbalance of this process of cognitive appraisal will constitute the magnitude of stress, which is perceived by the individual and consequently experienced as strain. Secondary appraisal assesses the type of coping options that are available, the probability that a particular option will be successful, and the capacity of the individual to successfully apply the strategy [9]. Results from secondary appraisals feed back to primary appraisals over time and attenuates the amount of stress experienced [10]. 

Executive appraisal occurs in the frontal cortex, which has extensive neural connections with the amygdala (stress activation pathway) and hippocampus (stress deactivation pathway) and is involved with the ultimate regulation of emotional expression at all times even during stress [11–13]. As distinct from other mammals and archaic man, the brain of modern humans is characterized by an extensive frontal cortex, comprising docosahexaenoic acid (DHA)-rich phospholipids [14]. 

Analysis of the fossil record has demonstrated that a turning point in the evolution of humans, between Neanderthal man and early modern humans from the Paleolithic period, was marked by the inclusion of seafood in the diet [15, 16]. The discovery of a rich and consistent source of marine long chain omega 3 polyunsaturated fatty acids (LCn-3PUFAs) is likely to have precipitated the evolution of the extensive cerebral hemispheres that characterizes the brain of modern humans [17, 18] and “rendered humans more resilient to natural pressures and the increasingly packed social environments of Late Pleistocene Europe” [16]. 

Humans have adapted to dietary LCn-3FAs, consuming equal amounts of n-3 to n-6FAs throughout evolution [19]. Industrialization has resulted in dramatic changes in the diet, with dramatic increases in the intake of n-6FA and reductions in n-3FA [20, 21]. The loss of n-3FAs from the diet corresponds with their loss from cell membrane phospholipids and with the overrepresentation of n-6FAs in the diet and cell membranes. LC-PUFAs in cell membranes act as substrates for bioactive inflammatory mediators that are activated during stress. N-6FA metabolites are proinflammatory, prothrombotic, and proaggregatory. LCn-3PUFAs mediate anti-inflammatory, proresolving, and antioxidant actions that maintain homeostasis during local tissue stress [22–25]. The loss of LCn-3FAs from the diet and cell membranes coincides with widespread stress and the rise of Western “lifestyle” diseases, characterized by chronic inflammation [26, 27]. 

There is little doubt that stress is an inflammatory state, mediated by proinflammatory cytokines and other inflammatory mediators [28–33], but a missing link to this knowledge is that inflammation is influenced by food consumption [34]. The immune system is intrinsically linked to the digestive system as food contains potential pathogens, which may invade and threaten homeostasis [35]. Local immune defenses in the gut are linked with systemic defense mechanisms in the blood in case a full immune response is required [36]. A range of inflammatory biomarkers, such as C-reactive protein and interlukin-6, have been found in postprandial bloods, but, interestingly, all foods do not elicit the same response. The three most contributing dietary factors predicting an exaggerated inflammatory response to a meal are the caloric value, glycemic index, and the lipid profile. The most critical lipid modulator is the amount of n-3 relative to n-6FAs in the diet and tissue, since at a tissue level n-3Fas suppress and n-6FAs promote postprandial inflammatory markers [37]. 

In an environment of chronic stress at work or financial stress, the consumption of foods which cause exaggerated inflammatory responses becomes another, subconscious, driver of the stress response. Chronic exposure to stressors, metabolic and/or psychosocial, predisposes towards low-level chronic metabolic or systemic inflammation, known as “metabolic dysregulation”, which has been implicated in the mediation of Western “lifestyle” diseases such as obesity, diabetes, and heart disease [35, 36]. 

Long-term overconsumption of foods rich in n-6FAs, such as processed foods containing generic vegetable oils, together with underconsumption of n-3FAs, from marine sources, not only exposes the body to an environment of chronic metabolic stress but also increases the proportion of n-6 to n-3FAs in cell membrane phospholipids [38, 39]. Cell membranes routinely release PUFAs to generate inflammatory mediators as part of a homeostatic response to any change in the cell membrane. At synaptic membranes, the liberation of LC-PUFAs (AA, EPA, and DHA) from the phospholipids upon stimulation by *PLA_2_* is coupled by G-proteins to numerous receptors, such that during routine cholinergic stimulation; LC-PUFAs are released from the membrane into the cytosol whereby small fractions of the substrates are converted into bioactive signaling molecules (eicosanoids or docosanoids) [40]. Most of the remaining released fatty acids are recycled back into the membrane phospholipids [41]. 

If n-6FAs are overrepresented, the signaling molecules generated are predominantly proinflammatory [40, 42]. If present in adequate proportions, LCn-3FAs balance the inflammatory impact of n-6FA metabolites by the generation of alternate families of much milder signaling molecules which are antagonistic to the n-6FA families and thereby effectively anti-inflammatory [43]. While overconsumption of n-6FA foods is rife, most Australians has are consuming just one-quarter of the LC n-3FAs (EPA and DHA) required to protect against chronic inflammatory diseases [44]. 

EPA and DHA have been progressively processed out of the Western food supply over the past century [45]. The loss of PUFA, particularly DHA, from our modern food was graphically illustrated in a set of studies by Crawford et al., who compared 12 matched samples of African buffalo and Western supermarket meat. In the Western meat, 97.8% of the total fatty acids were saturated and monounsaturated, and just 2.2% was essential polyunsaturated fatty acids (PUFAs), compared with 78.7% saturated and monounsaturated to 21.9% PUFAs in African meat [46]. 

Watson et al. [47] also demonstrated that meat from wild animals contains significantly higher DHA (0.7% venison; 0.9% in buffalo) than meat from domesticated animals, which contains only trace levels (<0.1%) regardless of whether from conventional or organic meat. Arya et al. [48] demonstrated a significantly exaggerated inflammatory response in the postprandial bloods of 10 volunteers after consumption wagyu, a modern type of hybridized beef, compared with wild game (kangaroo). 

The modern Western diet is a “proinflammatory diet” [49]. It is characterized by high caloric intake and high intake of refined carbohydrates, saturated, *trans-* and n-6 fatty acids, and low intakes of antioxidants and n-3 FAs. This study addresses this previously overlooked but yet critical link, the nutritional component of stress. Without evidence to inform our knowledge of the role of nutrition in stress, our ability to understand and deal effectively with stress is limited. This modeling aims to bridge a gap in the knowledge by linking stress outcomes with inflammatory markers and previously overlooked dietary markers. 

**Figure 1:**
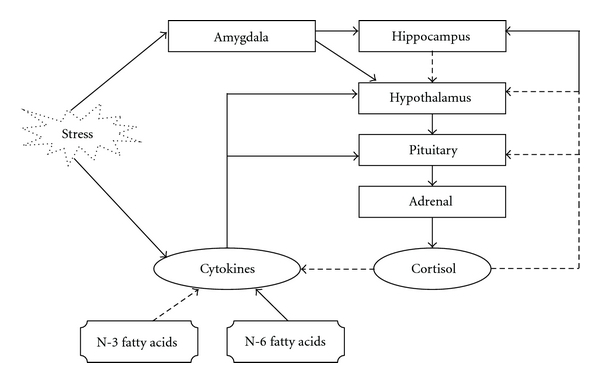
The hypothalamus-pituitary-adrenal axis, showing regulatory (dashed lines) and stimulatory (filled lines) influences.

Manipulation of dietary fats and the resultant reduction of the inflammatory impact of the diet during stress, as illustrated in Figure 1, are a novel approach to stress management. To the extent that metabolic stress is minimized, dealing with psychological stress may be more manageable. There has already been some promising research of the role of n-3FAs in stress [50–57]. This research aims to demonstrate that n-3FAs are critical for the balanced self-regulation (homeostasis) of both inflammation and stress outcomes. The research question was *what is the size of the impact of n-3FAs in stress and to what extent is it mediated via the suppression of proinflammatory cytokines?*

## 2. Materials and Methods

A cross-sectional study design was used for data collection, and structural equation modeling (SEM) was used to develop the statistical model. The minimum sample size for SEM is *n* = 200, based on 10 participants per parameter to be estimated. Participants were required to complete five self-report questionnaires and give a blood sample at one measurement point.

### 2.1. Statistical Modeling

Structural equation modeling (SEM) is an umbrella term for a family of related statistical techniques designed to represent complex relationships with parsimonious models. SEM has the capacity to incorporate multiple relationships between latent (unobserved) variables. It can be conceptualized as a combination between confirmatory factor analysis (CFA) (to estimate measurement models) and path analysis (to estimate structural relationships). One of its major advantages over other statistical techniques is that it tests multiple hypotheses simultaneously while taking into account all the error terms associated with all the variables [58]. By standardizing all variables (latent and observed), the path coefficients can be compared to assess the relative strengths of the relationships among a group of variables, while controlling for all the other relationships (regressions and correlations) in the model.

**Figure 2:**
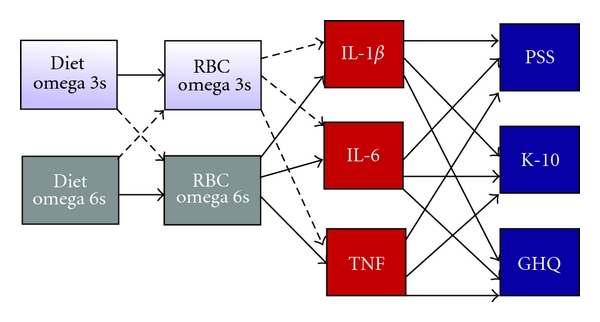
Path model for the hypothesized relationships between dietary intake of fatty acids, cell membrane fatty acids, proinflammatory cytokines, and several measures of psychological stress. Dashed lines represent negative regressions. RBC: red blood cell; IL: interleukin; TNF: tumour necrosis factor alpha; PSS: perceived stress scale; K-10: kessler 10 scale of psychological distress; GHQ: general health questionnaire.

A structural equation model can be drawn as a path diagram, such as that shown in Figure 2 with all the variables positioned to represent the relationships with other variables. The paths (arrows) represent the type of structural relationships (straight line is regression), and path coefficients are determined to represent the strength of the relationships. Standard errors are also estimated, so that the significance level for each path coefficient can be determined (that is if the critical ratio or *t* ≥ 1.96, then the parameter is significantly different from zero) [59]. 

The major advantage of SEM over path analysis and multiple regression, however, is the ability to analyze latent variables. Psychological constructs, such as “love”, “intelligence,” and “stress” by their very nature are difficult to define and measure. Psychological stress, for instance, is a complex multidimensional construct. It is made up of many factors, some of which are positive, such as successful coping ability. SEM is a multivariate methodology that can analyze models with multiple latent outcomes and multiple predictors. It can estimate complex relationships between latent constructs and has the capacity to treat them as both independent and dependent variables, enabling modelling of mediations. 

**Figure 3:**
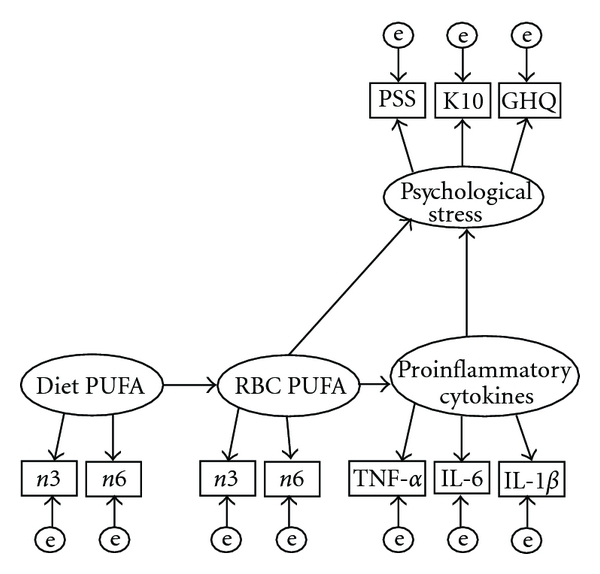
The *a priori* conceptualised partial mediation model for the relationships between the fatty acids and stress as mediated via proinflammatory cytokines. e-measurement error; PUFA: polyunsaturated fatty acids; TNF: tumour necrosis factor, IL: interlukin.

The formal sequence involved in SEM methodology is similar to confirmatory factor analysis (CFA) in that a model is first specified from theory or substantive knowledge. The data are then collected and the model tested. If the theoretically derived model fits the empirical data, then the model confirms the theory. The first step in specifying an *a priori* structural equation model is to formulate a model which best represents the relationships between variables based on theory and/or knowledge. This usually takes the form of a path diagram. When drawing such a diagram, the convention is to use an eclipse shape to denote a latent variable, a rectangle to represent an observed variable, and a circle to denote measurement error, also known as unique error variance (the variance not attributed to the factor). The *a priori* conceptualized structural model for the present research hypothesis is illustrated in Figure 3. 

Figure 3 shows the two different levels of measurement within the full SEM model. The “measurement models” are the latent factors and their respective indicators. The measurement model includes the factor loadings and the error terms associated with each item. The “structural model” incorporates the relationships between latent variables. The structural model includes the regressions between dependant variables, the regressions of the dependent variables on the independent variables, the factor variances, and the variances and covariances of structural disturbances or residuals (the amount of variance not explained by the model). 

During model testing, the formal hypothesis tested is the null hypothesis that there is *no significant difference* between the covariance structure estimated by the hypothesized model and that contained within the empirical data. A *nonsignificant* model chi-square statistic thus provides support that the *model is representative* of the structure of the relationships within the data. It should also be noted that there are some issues with the chi-square statistic such that, by consensus, it is not used on its own but as part of a battery of goodness of fit statistics (GOF), where a holistic assessment of “model fit” or how closely a model represents the structural relationships in the data should be taken.

Alternate models can be compared using the chi-square difference test for nested models. A model is “nested” within a more general model if it can be derived from the more general model by constraining one or more of its parameters, such as fixing the value of the parameter to be zero. A structural path (regression) is fixed to zero (not estimated) when there is no relationship between the variables joined by the path [60]. The chi-square difference and its associated degrees of freedom test the hypothesis that the two models are equivalent. If the chi-square difference is statistically significant (*P* < 0.5), then it is concluded that the two models are statistically different, and the constraints are not accepted. 

By tradition, if the models are found to be equivalent, the constraints are accepted due to the improvement in model parsimony. That is, the freeing of more degrees of freedom by constraining parameters results in a more parsimonious model. Parsimony has long been held as a highly desirable feature of statistical modeling. While the reasons for this may have philosophical roots, it can be statistically rationalized. With less parameters estimated, there are more degrees of freedom available for hypothesis testing. As more hypothesis testing may lead to more opportunities to falsify the hypothesis, more parsimony is considered the more scientifically rigorous approach [61]. 

The hypothesized model (Figure 3) states that dietary polyunsaturated fatty acids predict RBC membrane polyunsaturated fatty acids, which predict proinflammatory cytokines (*ex vivo* stimulated production of IL-1*β*, IL-6, TNF-*α*) which predict psychological stress levels (as measured by the PSS, GHQ-12, and K10). Specifically, membrane DHA would have inhibitory effects on proinflammatory cytokine production, which would reduce the inflammatory impact of the proinflammatory cytokines during stress and be reflected by lower levels of perceived stress. 

Preliminary univariate and multivariate statistical analyses were conducted using SPSS 18.0. Structural equation modeling (SEM) was conducted with Lisrel 8.80. All SEM used maximum likelihood extraction. Models were evaluated using the Satorra-Bentler “robust” chi-square test. A good model fit was reflected by a nonsignificant chi-square statistic (i.e., *P* > 0.05). Other goodness of fit (GOF) indices reflecting adequate model fit include the RMSEA <0.05, the goodness-of-fit index (GFI) and the adjusted goodness-of-fit index (AGFI) >0.90–0.95 [62], the non-normed fit index (NNFI) >0.95, and the standardized root mean square residual (SRMR) <0.8. 

The goodness-of-fit index (GFI) is the proportion of variance in the data covariance matrix accounted for by the model-estimated covariance matrix [62, 63]. This is the multivariate (*R* ^2^) equivalent of the bivariate coefficient of determination (*r* ^2^) [64]. The adjusted goodness-of-fit index (AGFI) takes into account the number of parameters (degrees of freedom) estimated in the model and punishes less parsimonious (over parameterized) models. The accepted range for these indices in models with a good fit is generally >0.90–0.95 [62]. 

The non-normed fit index (NNFI) is synonymous with the Tucker-Lewis Index (TLI) and Rho (**ρ**) and compares the model with a baseline model, usually the null, or independence, model where there are no significant relationships between variables, and all the regression coefficients are zero. Values should be >0.95 and values over 1 indicate the possibility of too many parameters in the model. The standardized root mean square residual (SRMR) is based on the differences (residuals) between the variances and covariances of the estimated covariance matrix and those of the sample covariance matrix. A perfect model fit will have an SRMR of zero. Values under 0.8 are considered to reflect an adequate model fit. 

To test the hypothesis that the effect of dietary polyunsaturated fatty acids on stress outcomes is mediated by proinflammatory cytokines, a mediation model such as that proposed (Figure 3) will be estimated. Kenny [65] defines: “The amount of mediation, which is called the *indirect effect*, is defined as the reduction of the effect of the initial variable on the outcome.” Baron and Kenny [66] have outlined four steps required to establish whether an effect on an outcome variable is mediated through a third variable.

- The independent/predictor variable is related to the dependant/outcome variable.
- The independent/predictor variable is related to the mediator variable.
- The mediator variable is related to the dependant/outcome variable, while controlling for the effects of the initial variable.
-
  - In complete mediation, the effect of the independent/predictor variable on the outcome variable is zero when the effect of the mediator variable is controlled.
  - In partial mediation, this effect is significantly reduced.

Kenny has recently cautioned that these steps should not be defined in terms of statistical significance alone as small sample sizes may find large effect sizes nonsignificant [65].

### 2.2. Outcome Measures

#### 2.2.1. Subjective

*The perceived stress scale (PSS-10) *[67] is a validated and reliable subjective measure of the amount of psychological or emotional stress an individual perceives. The PSS is made up of ten items which are scored from 0–4. The items ask about the frequency of occurrences of events from “never” to “very often”. Four of the items are positively worded, asking about available coping resources, and are reversed scored. *The Kessler-10 (K-10)* [68] has been shown to have strong accuracy in predicting those at risk of mental health disorders in the Australian population [69]. *The general health questionnaire (GHQ-12)* [70] is a valid and reliable measure, routinely used in stress research. *The food frequency questionnaire (FFQ)* [71] is a validated self-report questionnaire and is analysed by the Cancer Council using a version of Xyris software that incorporates the RMIT Fatty Acid Database of Australian Foods to enable analysis of individual polyunsaturated fatty acids. *A social history questionnaire* was administered to measure possible covariates, such as physical exercise level, coffee intake and hours of sleep within the 72 hours prior to the study.

#### 2.2.2. Objective

*Proinflammatory cytokine production:* monocyte secretions of cytokines in whole blood were assessed after blood was drawn and stimulated. Whole blood was stimulated *ex vivo* with lipopolysaccharide (LPS) from *E. coli*. Culture was incubated for 4 hours at 37°C and the supernatant harvested and frozen at −80°C. At the end of data collection, all samples were analysed as a single batch. Cytokines were quantified by Becton-Dickenson methods involving bead array cytokine testing following the protocol as provided by the cytometric bead array (CBA) human inflammation kit. *RBC fatty acid analysis: *approximately 2 mLs of the blood sample was drawn for the red blood cell (RBC) fatty acid analysis. Packed RBCs were stored at −80°. At the end of data collection, samples were transferred as a batch to Wollongong University for extraction and analysis. Total phospholipid fatty acids were extracted from erythrocyte membranes and analysed using gas chromatography (GC). The fatty acids of interest were the long chain omega 3 and omega 6 polyunsaturated fatty acids, particularly EPA, DHA, and arachidonic acid. The protocol the laboratory followed was previously published by Lepage and Roy [72].

### 2.3. Participants and Study Procedure

The sample was drawn from the general population of healthy adults over 18 years of age. The exclusion criteria were diabetes, heart disease, inflammatory diseases, and regular use of anti-inflammatory medications. 

Participants responded to promotion and advertisements by phone or email to arrange a time to attend the clinic after an initial phone screen. There were no remunerations for participation but participants were provided with a complimentary breakfast after the blood tests had been taken. In the clinic participants completed five questionnaires and undertook anthropometric assessments. Participants were asked to fast and avoid strenuous exercise and medications such as paracetamole for 12 hours prior to giving blood and to attend the clinic between 8–10 am on a prearranged day. Two blood samples of 4 mLs were drawn from a single venipuncture. The anticoagulants used were heparin for the cytokine sample and EDTA for the FA sample.

## 3. Results and Discussion

### 3.1. Sample Characteristics

The sample consisted of 194 adults aged between 18 and 69 years, with a mean age of 33.8 years (standard deviation (SD) = 12 years). The proportion of women was far greater than men, with 143 (74%) females and 51 (26%) males. The sample was recruited from two major centers, Southern Cross University, a regional university based in Lismore, northern NSW (55% of the sample), and St Lucia, the Brisbane campus of the University of Queensland (43% of sample). Most (94.9%) of the cohort was drawn from the population of university staff and students.

### 3.2. Preliminary Analysis

Many of the variables used in the study were not normally distributed and were significantly skewed and kurtotic. The distribution skews were generally to the right, indicating that most people scored lower but a few had very high scores. This was to be expected for the distress scales (K-10 and GHQ-12) and for the blood samples of essential fatty acids and proinflammatory cytokine secretion. Extreme multivariate outliers were identified and removed from the analyses prior to modeling. The number of multivariate outliers removed from each analysis is noted in the respective model figure legends. 

The erythrocyte phospholipid fatty acids were expressed both in absolute terms (used in the modeling) and as a percentage (or fraction) of total fatty acids present in the membrane. Arachidonic acid was the most dominant polyunsaturated fatty acid in cell membrane phospholipids (13.592 (SD 1.313) mols% of total fatty acids). This was followed by linoleic acid at 9.645 (1.297) mols% of total fatty acids. The omega 3 fatty acid with the highest mean ratio was DHA with 4.263 (1.051), followed by DPA with 2.236 (0.396) mols% total fatty acids. EPA had a mean proportion of just 0.56 (SD 0.67) mols% of total fatty acids. The ratio of omega 6 to omega 3 polyunsaturated fatty acid fractions was 4.203 (SD 1.26) mols% total fatty acids. In absolute amounts, the sample mean for arachidonic acid was 35.715 (SD 14.043) *μ*g/mL and linolenic acid was 23.466 (9.906) *μ*g/mL. The third highest fatty acid was DHA with a sample mean of 12.111(5.478) *μ*g/mL, followed by the DPAn-3 with 6.407 (2.733) *μ*g/mL. EPA had a sample mean of 1.584 *μ*g/mL but its standard deviation was higher than its mean, at 1.793 *μ*g/mL, signaling potential measurement issues. 

EPA was present in very small quantities in erythrocyte membranes. In almost half the sample (47.9%) the levels of EPA were below the limit of detection of the gas chromatographer (GC) and were not reliably measured by the laboratory outsourced to conduct the analysis. The ensuing loss of data was dealt with statistically by multiple imputation (MI), although EPA remained a problem variable during modeling and was eventually withdrawn from analysis.

### 3.3. Modeling Dietary Fatty Acids with RBC Membrane Fatty Acids

The dietary fatty acids, as measured by the FFQ, were not reliable predictors of RBC fatty acids. In the omega 3 model (which tested the hypothesis that FFQ EPA + DHA predicts RBC EPA + DHA), a proper solution could not be estimated due to serious colinearity issues between FFQ EPA and FFQ DHA. That is, the correlation between them was so high (0.999) that statistically they were treated as the same thing. This suggests that the database which estimated these values could not adequately distinguish between EPA and DHA. To the degree that useful information could be gained from these models using the FFQ data, there was little evidence of reliable relationships between dietary polyunsaturated fatty acids and red blood cell polyunsaturated fatty acids. For these reasons the decision was made to leave out the FFQ variables from the final mediation modeling.

### 3.4. Modeling RBC Membrane Fatty Acids with Cytokines

Modeling the RBC fatty acid with cytokines was initially problematic due to the imputed EPA variable, as previously described. EPA (as imputed) was not a reliable variable (squared multiple correlation of 0.29 with the latent omega 3 factor), suggesting that 70% of the variation in EPA was error variance (variance not explained by the omega 3 factor) and the inclusion of EPA consistently caused modeling difficulties, such as convergence problems and solutions with large standard errors causing unstable parameter estimates. Membrane EPA was thus deemed an unreliable variable in the current data and was withdrawn from further modeling.

Modeling with the fatty acids thus proceeded by using the omega 6 fatty acid, arachidonic acid (AA), the omega 3 fatty acid, and docosahexaenoic acid (DHA), as two independent predictors of stress outcomes (direct effects) and as mediated via influencing the production of proinflammatory cytokines (indirect effects). 

### 3.5. Modeling Proinflammatory Cytokines and Psychological Stress

**Figure 4:**
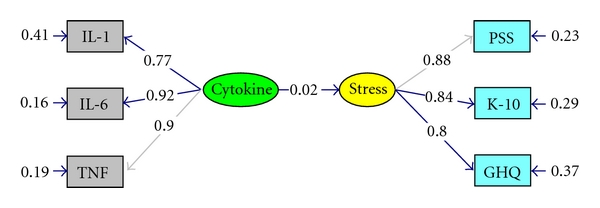
Basic model for global stress and cytokines. *χ* ^2^ = 3.86, *df* = 8, *P* = 0.86992, RMSEA ≤ 0.0005; Five multivariate outliers excluded (*n* = 189); all parameters fully standardized.

The global measure for psychological stress, as measured by the total scores of three different stress scales, was not significantly regressed on the proinflammatory cytokines factor, as shown in Figure 4. 

The model was a good fit to the data, as indicated by the nonsignificant chi-square statistic. This was further corroborated by the GFI = 0.99, AGFI = 0.98, and the NNFI = 1.01. All the parameters in the model (Figure 4) were significant, with the exception of the regression of STRESS on CYTOKINE (0.02, *t* = 0.31). The SMC (squared multiple correlation, or amount of explained variance) for the latent variable STRESS was 0.0006, and the structural equation residual was 0.9994, indicating that the only 0.06% of the variance in STRESS was accounted for by its regression on CYTOKINE, while almost all of its variance (99.94%) was unaccounted for by the model. 

It would appear from this model and the correlations shown in Table 1 that there were no relationships between stress and cytokines. 

CFA (confirmatory factor analysis), however, had previously indicated that each scale consisted of several subscales. At that stage it had been hypothesized that the biomarkers would probably relate differently to each of the subscales, particularly as the subscales represented both positive (such as “coping”) and negative (such as “strain”) aspects of stress appraisal. To test this hypothesis, the subscales were included in the model as more specific measurement models for stress. 

Previous modeling with CFA on the PSS-10 had confirmed that the scale comprised three factors or subscales: Coping (items 4, 7, 5), Overwhelm (2, 10, 6 and 8), and Emotional Reactivity (1, 9 and 3). (The three-factor confirmatory model for the PSS-10 model fit statistics: *χ* ^2^ = 40.17, *df* = 32, *P* = 0.15209, RMSEA = 0.036). The coping factor was negatively correlated with the two other strain-related factors. Of particularly interest was that the two strain-related factors (Overwhelm and Emotional Reactivity) were equal contributors to the higher-order construct (Perceived Stress), but the Coping factor was a significantly stronger predictor (this modeling is detailed in a separate paper). 

**Figure 5:**
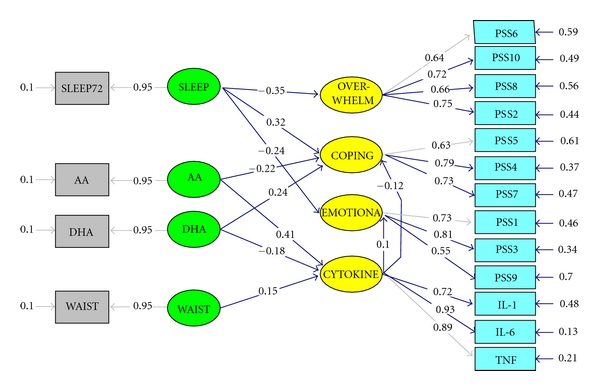
Structural model for partial mediation of the Fatty Acids on PSS Coping via CYTOKINE. *χ* ^2^ = 144.66, *df* = 108, *P* = 0.01068, RMSEA = 0.042; GFI = 0.92; AGFI = 0.88; NNFI = 0.98; SRMR = 0.053; Four multivariate outliers excluded (*n* = 190); All parameters fully standardized; significant pathways indicated by asterix in Table 3. PSS: Perceived Stress Scale; SLEEP: number of hours sleep in the 72 hours prior to the blood draw; AA: RBC membrane arachidonic acid, as measured in absolute amounts (*μ*g/mL); DHA: RBC membrane docosahexaenoic acid, as measured in absolute amounts (*μ*g/mL); WAIST: waist circumference; OVERWHELM: latent factor representing PSS questions 6, 10, 2, and 8, where 8 was negatively loaded; Coping: latent factor representing PSS questions 7, 5, and 4; EMOTIONAL: latent factor representing PSS questions 1, 3, and 9. Curved arrows represent correlations, straight arrows represent regressions, arrows pointing in to outcome variables represent structural disturbances (errors).

To the extent that one subscale contributes differentially to the higher order factor, but it is treated as if it contributes equally (by summing across the items, giving each item the same weight), the unreliability of the model is increased. By treating the indicators all as equal contributors, the effects of coping are diluted and the model loses sensitivity. Once it was discovered that the coping scale was a significantly stronger predictor than the other two subscales in the PSS it was decided that it would be more accurate to model the PSS as its three subscales rather than use the higher-order construct, “Perceived Stress”, represented by the total score. The three subscales (labeled “Emotional”, “Coping”, and “Overwhelmed”) were modeled as such with the proinflammatory cytokines (labeled Cyotkine), fatty acids (labeled AA and DHA, resp.) and significant covariates (number of hours sleep in the past 72 hours (labeled SLEEP) and waist circumference (labeled WAIST) in a mediation model as illustrated in Figure 5. 

A preliminary correlation analysis of the relationships between the stress factors (computed by averaging across the indicators within each factor) and cytokine composite variables, provided in Table 2, shows that when grouped together by averaging, the three proinflammatory cytokines were negatively correlated with the COPING factor, although not significantly (*r* = −0.10, *P* = 0.17) and positively but not significantly associated with the factor labeled Emotional (*r* = −0.06, *P* = 0.41). It is also evident from Table 2 that the three stress factors are moderately but not too highly correlated with each other, indicating that the three factors represent three different but related factors. 

 The discriminate validity shown between the factors of the PSS supports the notion of representing the factors separately in further modeling. By representing the factors separately in a structural equation model, each factor is free to relate differentially to the other variables in the model. Therefore, representing “perceived stress” instead as its three constituent factors has the potential to more closely represent the relationships between the variables in the data. 

As a further preliminary exploratory step, separate regression analyses on the relationships hypothesized to form the mediation model were sequentially analyzed. These results have been combined and presented in Table 3. 

The only statistically significant coefficient presented (Table 3) was the regression of the dependant variable CytokineAve on the independent variable arachidonic acid (*t* = 3.085, *P* = 0.002). This relationship remained significant even after post-hoc adjustment to the significance levels to account for multiple (three models) tests (*P* = 0.05/3 = 0.02). 

Kenny [65], however, advises not to discount mediation effects based on nonsignificance alone, as *P *values are influenced by small sample sizes. The fact that the other relationships are not far from significance in these models (Table 3) suggests that a more accurate modeling methodology, one that has the potential to partial out error variances from the composite (latent) variables, may provide more reliable estimates of the structural relationships (regressions). Therefore the hypothesized structural relationships were tested in a structural equation model. 

The full mediation structural equation model (Figure 5) was not an excellent fit to the data, as indicated by a significant robust chi-square test statistic (*χ* ^2^ = 144.62*df* = 108, *P* = 0.01074, RMSEA = 0.042). However the RMSEA and other GOF indices indicate that the model was not an unreasonable approximation of the data (GFI = 0.92, AGFI = 0.88, NNFI = 0.98, SRMR = 0.053). The adjusted goodness of fit index (AGFI) was a little low, but all other fit statistics were within the acceptable ranges. Together with the chi-square and RMSEA, these statistics indicate that the model was an adequate representation of the data. 

The SMC for the Coping indicated that 14% of its variance was accounted for by the model. In this model, Coping was significantly regressed on (predicted by) DHA (0.24, *t* = 2.05), indicating that for every standard deviation increase in DHA there is 24% of one standard deviation increase in the scores for the PSS subscale Coping. There was a trend towards a significant negative regression of Coping on AA (−0.22, *t* = −1.83), indicating that for every one standard deviation increase in arachidonic acid there is a 22% of one standard deviation reduction in scores on the PSS subscale Coping. 

Coping was negatively regressed on CYTOKINE (unstandardized −0.05, SE = 0.03, *t* = −1.66), while Emotional was positively regressed on CYTOKINE (unstandardized 0.06, SE = 0.04, *t* = 1.45). The standardized regression coefficients are given on the paths in the model (Figure 5). They indicate that for every standard deviation increase in cytokine production, there is 10% of one standard deviation increase in EMOTIONAL reactivity and 11% of a standard deviation reduction in Coping scores.

CYTOKINE was significantly and positively regressed on AA (0.42, *t* = 2.30), indicating that for every standard deviation increase in arachidonic acid, there is 42% of a standard deviation increase in cytokine production. CYTOKINE was negatively nonsignificantly regressed on DHA (−0.18, *t* = −0.98), indicating that for every standard deviation increase in DHA there is 18% of a standard deviation reduction in cytokine production. 

In this partially mediated model (Figure 5) all the pathways in the hypotheses were estimated. To measure the extent to which the effects of the fatty acids on PSS-Coping were mediated via the proinflammatory cytokines, the *total* and *indirect effects* were compared and are shown in Table 4. There were significant total effects for the regressions of PSS-Coping on both arachidonic acid and DHA. The indirect effects of the fatty acids on PSS-Coping were much less substantial and nonsignificant.

The proportion of indirect effect to total effect for AA was 18.19%. This is the amount of mediation effects on PSS-Coping via stimulating the production and secretion of proinflammatory cytokines; the direct effects (81.81%) were much more important. Similarly, the proportion of the effect of DHA on PSS-Coping that was mediated through the inhibition of the secretion of proinflammatory cytokines was 7.69%, indicating that 92.33% of its total effect was a direct effect. 

Together these findings demonstrate that the effects of the essential fatty acids on PSS-Coping were predominately direct effects and were not significantly mediated via influencing the proinflammatory cytokines. There was a tendency towards partial mediation of the effects of arachidonic acid on PSS-Coping via stimulating the production of proinflammatory cytokines. To formally assess whether the partially-mediated model was the best representation of the data, it was compared with two alternate models: (i) the nonmediated model and (ii) the completely mediated model. 

### 3.6. Mediation Modeling

In the *direct effects* or nonmediated model (Table 5, model A), the effects of the fatty acids on Coping were not mediated via the cytokines, such that the paths from the respective fatty acids (AA and DHA) to CYTOKINE were fixed to zero to reflect no relationship. This model was a statistically significant worse model than the partially mediated model. The chi-square difference test for nested models (Table 5, model A—model C) was significant (*P* = 0.008). The significant increase in chi-square after the removal of the direct effects paths suggests model misspecification. Therefore the nonmediated model was not accepted as adequately representative of the relationships within the empirical data. 

Having established that some form of mediation best represents the relationships within the data, the fully mediated model was tested and compared with the partially mediated model. In the *indirect effects* or completely mediated model (Table 5, model B), the direct effects of the fatty acids (AA and DHA) on Coping were not estimated (fixed to zero), and all effects of the respective fatty acids were mediated via CYTOKINE. When fitted to the data, this completely mediated model was not an unreasonable fit to the data and was comparable to the partially mediated model, according to its chi-square test statistic, as given in Table 5 (model B) and other GOF statistics (RMSEA = 0.042, GFI = 0.92, AGFI = 0.88, NNFI = 0.98, SRMR = 0.053). 

The decision whether the structural relationships in the data are best represented by a completely mediated or partially mediated model should be determined by both theoretical and statistical considerations. Although the completely mediated model appears to be more parsimonious (less parameters and more degrees of freedom), we know that only a small proportion (20—25%) of the effects of the fatty acids on coping were mediated via proinflammatory cytokines. Therefore, the model which best represents both the theory and the data is the partially mediated model.

### 3.7. Summary of Findings

The hypotheses were tested with a mediation model. The direct effects of the fatty acids on stress were compared with the indirect effects, which were mediated via proinflammatory cytokines. A mediation model could not be established for global stress, as it was not significantly regressed on the proinflammatory cytokines. Global stress, as indicated by the total scores of the three stress and distress scales, was found to lack sufficient specificity to effectively represent the construct of stress, as originally set out in the *a priori* conceptual model. Factor analysis on each of the stress scales had consistently demonstrated that stress was a multidimensional construct, and its representation as a single global factor did not capture the subtly differentiated relationships between the various stress factors, proinflammatory cytokine production, and membrane phospholipid fatty acids.

When stress was instead modeled as a multi-dimensional construct, a mediation model was established for the PSS factor “coping”. The model was a partially mediated model, whereby all the relationships were estimated; coping was regressed on the fatty acids, as well as proinflammatory cytokines, which in turn were regressed on the fatty acids. From this partially mediated model, direct and indirect (mediated) effects could be estimated. These data would provide the answer to the research question regarding the size of the impact of fatty acids on stress (coping) and the extent to which these effects are mediated via proinflammatory cytokines. 

Mediation models were sought from all three stress scales. The best mediation model used the three-factor confirmatory PSS model as the measurement model for stress. The latent variable, “coping”, reflected a positive state and was negatively related to two negative state stress factors, “overwhelm” and “emotional”. In this three-factor model of the perceived stress scale (PSS), each latent variable had at least three indicator variables. 

This factorial structure of the PSS satisfied the identification rules for modeling with latent variables (each latent variable requires at least three indicators) and may be the reason that this model was the most successful of the three stress scales for this research question. The factor structures of both the GHQ-12 and the K-10 contained two-indicator latent variables that, when combined with a small sample size and minor deviations from other assumptions such as normality and linearity, were associated with increased frequency of modeling methodological issues such as nonconvergence and improper solutions. 

It was hypothesized from the outset that cytokines would directly predict global stress. The data did not support this theory. The effect of the proinflammatory cytokines on global stress was negligible. On further investigation, however, the underlying relationships were far more subtle and complex than had been originally hypothesized. When stress was modeled instead as a multidimensional construct, it was found that the cytokines were related differentially to the different factors. The proinflammatory cytokines, as a group, were inversely related to “coping” but directly related to “emotionally reactive”. 

In the final partially mediated model with the PSS, the omega 3 fatty acid, DHA had a significant predictive relationship with coping. It was also demonstrated that only a small proportion (approximately only 7%) of this effect was mediated through the inhibition of proinflammatory cytokine production. Arachidonic acid was found to inversely predict coping. This effect was partially mediated via proinflammatory cytokine production. The proportion of arachidonic acid that was mediated via the stimulation of proinflammatory cytokine production was approximately 18% of the total effect of arachidonic acid on coping.

This modeling suggests that DHA is a stronger predictor of coping factors than of strain-related constructs. That is, DHA may play a beneficial role in helping to adapt to stress through assisting the coping mechanism. This finding is exciting and worthy of further investigation as it suggests that nutrition may have a direct role in coping with—or adaptation to—stress. Individuals vary widely in regards to coping (behavioral adaptation). Perhaps the most cutting edge research in the field of nutrition-facilitated adaptation is its potential to play a strategic role in the building of resilience in soldiers. A workshop was convened in the US last year entitled *Nutritional armor for the warfighter: can omega-3 fatty acids enhance stress resilience, wellness, and military performance?* to provide the US Department of Defense with an overview of the science behind n-3FAs in stress [73]. 

The recognition that n-3FAs may improve resilience for soldiers echoes the observations made of the early humans that the discovery of seafood may have helped to increase resilience to natural pressures. This work should be carried forward in studies that aim to demonstrate a causal relationship between n-3FAs and coping to include various coping outcomes and widening the scope of inflammatory markers in a longitudinal design. The findings from this study and other such studies should inform a deeper understanding of a valid role for nutrition, particularly the n-3FAs, in the building of resilience in populations.

### 3.8. Methodological Issues

Fatty acids were predictive of the secretion of cytokines in the hypothesized directions; arachidonic acid was a positive predictor, and the n-3 fatty acids were negative predictors of the production of proinflammatory cytokines. This was consistent with the theory and the weight of evidence [74–78], upon which the *a priori* hypothesized model was based. However, the predictive relationships of the fatty acids on cytokines and of cytokines on stress were not as substantial or significant as anticipated.

Methodological limitations including the loss of data for EPA during fatty acid analysis together with modeling limitations such as ordinal data in the PSS subscales in a relatively small sample may have been partially responsible for these outcomes. However, it is also possible that the relationships between DHA, arachidonic acid, proinflammatory cytokine production, and coping may contain interactions and that a more complete model may be estimated using nonlinear methodologies. The determination of curvilinear functions may more closely represent these relationships.

### 3.9. Future Modeling

Structural equation modeling is a powerful analytical technique that allows for the simultaneous testing of multiple hypotheses representing complex relationships between constructs. Humans are highly complex organisms, both psychologically and physiologically. SEM methodology seems remarkably well-suited to test theories of how dietary and immune parameters relate to latent psychological outcomes. There is a variety of SEM software available. This study used Lisrel, a traditional linear modeling technique. Future studies should consider using MPlus, as it readily analyzes both linear and nonlinear relationships. 

The findings from this research indicate that the polyunsaturated fatty acids have differential effects on stress and coping outcomes, which are partially mediated via the proinflammatory cytokines. The basic predictive structural relationships were confirmed as hypothesized, but only after some respecifications to the measurement models. The modifications to the measurement model for “stress” have highlighted the difficulties in modeling complex latent constructs. Future modeling should “unpack” multi-dimensional constructs prior to modeling with a view to establishing different models for each outcome. This process was followed here by breaking “perceived stress”, as measured by the PSS-10, into its three component factors. Of the three, the coping factor had the strongest relationships with the fatty acids and cytokines, facilitating mediation modeling. 

Future modeling in this area would ideally account for more of the variance in the outcome variables, stress, coping, and cytokines. This may be achieved by including other biomarkers for stress and cytokines, such as cortisol or broadening the scope of inflammatory markers, such as to include C-Reactive Protein or nuclear factor-*kappa* B. In addition, other covariates of coping and cytokines, such as tangible support, as found by Miller et al. [79] may be help explain more variance in these outcomes and give more explanatory power to the model. There may also be time effects that were not modeled here that could be controlled in future studies [80]. For instance, the effects of acute and chronic stress may have differential effects on proinflammatory cytokine production [81]. Finally, nonlinear modeling methodologies should explore the potential role of arachidonic acid as a moderator for the effects of the omega 3 fatty acids on cytokines and coping outcomes.

## 4. Conclusions

There is good evidence in the literature to suggest that omega 3 fatty acids may play a beneficial role during stress and that a potential mechanism is via the regulation of the production of proinflammatory mediators. This study sets out to test these hypotheses and measures the strength of the relationships. The most appropriate available methodology for the simultaneous testing of multiple hypotheses is structural equation modeling. Modeling in this sample demonstrated that the hypothesized pattern of relationships was supported, but the effect sizes were not as significant as anticipated. However, DHA had a significant direct effect on the Coping subscale from the PSS-10, which supports the research hypothesis in finding an adaptogenic role for DHA in stress. 

The effects of DHA on the PSS-Coping subscale were largely direct effects as only approximately 7% of this effect was mediated via the inhibition of proinflammatory cytokine production. Future modeling should explore whether adding EPA to the model would increase the significance and impact of these mediation pathways. As anticipated, the role of arachidonic acid was opposite to that of DHA, having a negative impact on PSS-Coping scores. The negative effect of arachidonic acid on coping was partially mediated (approximately 18%) via increasing the production of proinflammatory cytokines. 

This was the first attempt to model the relationships between essential fatty acids and immune activation biomarkers with subjective stress outcomes. It must be noted that this study used a linear modeling technique. Future modeling should consider that these relationships may not be linear and that nonlinear modeling methodologies may provide more powerful estimates of effect sizes.

## Figures and Tables

**Table 1:** Pearson moment correlations for stress and cytokine variables.

	PSS	K10	GHQ	IL-1	IL-6	TNF
PSS	1	0.720**	0.697**	0.044	0.053	0.017
K10		1	0.703**	0.007	0.032	0.024
GHQ			1	0.067	0.060	0.044
IL-1				1	0.675**	0.672**
IL-6					1	0.854**
TNF						1

*n *= 194; **correlation significant at the *P* ≤ 0.0005 level (two-tailed).

**Table 2:** Pearson moment correlations for PSS subscales and cytokine composite variables.

	OverwhelmAve	CopingAve	EmotionalAve	CytokineAve
OverwhelmAve	1	−.631**	.612**	−.010
CopingAve		1	−.605**	−.098
EmotionalAve			1	.060
CytokineAve				1

*n* = 194; composite scores computed by averaging across items in respective factors (unit-weighted); **Correlation significant at the *P* ≤ 0.0005 level (two-tailed).

**Table 3:** Three regression models testing for mediation effects.

		Independent variable	Beta	*t*	Sig.
*Direct effects*	Model 1	AA	−0.185	−1.715	0.088
	Dependent Variable: CopingAve	DHA	0.127	1.176	0.241

*Indirect (mediated) effects*	Model 2	AA	0.326	3.085	0.002
	Dependent Variable: CytokineAve	DHA	−0.169	−1.601	0.111
	Model 3				
	Dependent Variable: CopingAve	CytokineAve	−0.098	−1.365	0.174

*n *= 194; composite score for CytokineAve and CopingAve computed by averaging across items in respective factors (unit-weighted); AA = red blood cell membrane phospholipid arachidonic acid, measured in micrograms per millilitre of blood (*μ*g/mL); DHA = red blood cell membrane phospholipid docoxahexaenoic acid, measured in micrograms per millilitre of blood (*μ*g/mL).

**Table 4:** Standardized total and indirect effects of the fatty acids and covariates on the perceived stress scale (pss) subscales and cytokines.

	SLEEP	WAIST	AA	DHA
*Total effects*				
PSS-Overwhelm	−0.35*	—	—	—
PSS-Coping	0.32*	−0.02	−0.27*	0.26*
PSS-Emotional	−0.24*	0.01	0.04	−0.02
Cytokine	—	0.15	0.41*	−0.18

*Indirect effects (mediated effects)*				
PSS-Coping	—	−0.02	−0.05	0.02
PSS-Emotional	—	0.01	0.04	−0.02

*Direct effects (non-mediated effects)*				
PSS-Overwhelm	−0.35*	—	—	—
PSS-Coping	0.32*	—	−0.22	0.24*
PSS-Emotional	−0.24*	—	—	—
Cytokine	—	0.15	0.41*	−0.18

*Significant at *t* ≥ ±1.96 level. SLEEP: number of hours sleep in the 72 hours prior to the study; AA: RBC membrane arachidonic acid, as measured in absolute amounts (*μ*g/mL); DHA: RBC membrane docosahexaenoic acid, as measured in absolute amounts (*μ*g/mL); WAIST: waist circumference; PSS-Overwhelm: latent factor loading PSS-10 questions 6, 10, 2, and 8, where 8 was negatively loaded; PSS-Coping: latent factor loading PSS-10 questions 7, 5, and 4; PSS-Emotional: latent factor loading PSS-10 questions 1, 3, and 9.

**Table 5:** Model statistics for three alternative mediation models.

	Model			Chi-square difference test			
Hypothesis	Chi-Square	*df*	*P*		Chi-Square	*df*	*P*
A: Nonmediated Model	154.35	110	0.003				
B: Fully mediated Model	147.35	110	0.010	A-B	7	0	n/a
C: Partially mediated	144.66	108	0.011	A–C	9.690	2	0.008*
				B-C	2.690	2	0.261

*denotes significance at the *P* < 0.01 level.

**Table 6:** 

	Never	Almost never	Sometimes	Fairly often	Very often
(1) In the last 30 days, how often have you been upset because of something that happened unexpectedly?	0	1	2	3	4
(2) In the last 30 days, how often have you felt that you were unable to control the important things in your life?	0	1	2	3	4
(3) In the last 30 days, how often have you felt nervous and “stressed”?	0	1	2	3	4
(4) In the last 30 days, how often have you felt confident about your ability to handle your personal problems?	4	3	2	1	0
(5) In the last 30 days, how often have you felt that things were going your way?	4	3	2	1	0
(6) In the last 30 days, how often have you found that you could not cope with all the things that you had to do?	0	1	2	3	4
(7) In the last 30 days, how often have you been able to control irritations in your life?	4	3	2	1	0
(8) In the last 30 days, how often have you felt that you were on top of things?	4	3	2	1	0
(9) In the last 30 days, how often have you been angered because of things that were outside of your control?	0	1	2	3	4
(10) In the last 30 days, how often have you felt difficulties were piling up so high that you could not overcome them?	0	1	2	3	4

## Appendix

### The Perceived Stress Scale-10 Items

Instructions: the questions in this section ask you about your feelings and thoughts during the last month. In each case, please indicate how often you felt or thought a certain way (see Table 6).
